# Breastfeeding and human milk bank in a neonatal intensive care unit: impact of the COVID-19 pandemic in an Italian cohort of very low birth weight infants

**DOI:** 10.1186/s13006-022-00529-x

**Published:** 2022-12-29

**Authors:** Ilia Bresesti, Laura Morlacchi, Caterina Cazzaniga, Camilla Sangiorgio, Lorenza Bertù, Maria Elena Bolis, Angela Bossi, Massimo Agosti

**Affiliations:** 1 Division of Neonatology, Woman and Child Department, “F. Del Ponte” Hospital, ASST-Settelaghi, University of Insubria, 21100 Varese, Italy; 2 Department of Medicine and Surgery, University of Insubria, Varese, Italy

**Keywords:** Preterm infants, Neonatal nutrition, Donated human milk, Parental stress, COVID-19 pandemic

## Abstract

**Background:**

Parental stress in neonatal intensive care units (NICU) is well known, as is the stress induced by the COVID-19 pandemic. This combination might increase stress to the extent of affecting the availability of maternal expressed milk and the success of establishing breastfeeding. This is particularly relevant in very preterm infants.

**Methods:**

We conducted a single-centre retrospective analysis in two cohorts of very low birth weight infants born in a hospital in Italy. Babies born before the pandemic (September 2017 – December 2019) (*n* = 101) and during the pandemic (March 2020 – December 2021) (*n* = 67) were included in the analysis. We compared the rate of babies fed with maternal milk (both expressed and / or donated) at the achievement of full enteral feeding and the rate of those exclusively breastfed at discharge in the two groups. Then, we analysed the impact of donated human milk availability on infant formula use. We also compared mother’s need for psychological support during NICU stay and the duration of psychological follow-up after discharge.

**Results:**

In our NICU the availability of expressed maternal milk significantly decreased during the COVID-19 pandemic (86.1% before the pandemic vs 44.8% during the pandemic, *p* < 0.001) at the time of full enteral feeding achievement. Thanks to the availability of donated human milk, the rate of formula-fed babies remained almost unchanged (13.9% vs 14.9%). At discharge, the rate of breastfeeding was similar (73.3% vs 72.7%). The maternal need for psychological support was significantly higher during the pandemic (33% vs 64%, *p* < 0.001), as well as the duration of follow-up > 6 months (1% vs 15%, *p* < 0.001). No differences in the main clinical outcomes were found.

**Conclusion:**

Pandemic-induced stress had a significant impact on the availability of expressed maternal milk in NICU. However, the presence of human donated milk was fundamental in preventing increased use of infant formula during NICU stays. This underlines how strategies to implement the widespread establishment of donor milk banks on a national level are warranted. Further research is desirable to optimise the use of donated human milk banks during emergency situations.

## Background

Maternal milk is the first feeding choice to improve the short- and long-term outcomes of all neonates, especially for preterm and very low birth weight (VLBW) infants [1]. It promotes the achievement of early full enteral feeding (FEF), thus reducing complications linked to prolonged parenteral nutrition. It is also protective against the major morbidities affecting preterm infants, such as sepsis, necrotising enterocolitis and bronchopulmonary dysplasia [2, 3].

For these reasons, the use of expressed maternal milk is highly recommended in neonatal intensive care units (NICU). However, it is not uncommon that maternal milk is scarce, especially at an early stage. When it is not available or is insufficient, the use of donated human milk is recommended [4, 1]. Donated human milk provides several of the advantages of maternal milk, although it is associated with a significant risk of poor growth. Therefore, donated human milkis often combined with fortifiers to overcome this limiting factor [5, 6].

The successful establishment of breastfeeding in the neonatal intensive care unit (NICU) is highly related to the environmental conditions and to the support the mothers are given. The NICU setting, in fact, is known to be highly stressful for parents [7–9].

During the COVID-19 pandemic, the maternal stress related to recovery in NICU has markedly increased [10, 11]. Besides restrictions in daily life activities, also the routine management of NICUs has witnessed substantial changes, and strict limitations in visiting babies have been introduced in a large proportion of neonatal units worldwide [12, 13].

This study aimed to verify the impact of the COVID-19 pandemic on breastfeeding in the NICU and the role of a donor human milk bank in a cohort of VLBW infants.

## Methods

This retrospective study was conducted at the Neonatal Intensive Care Unit of the “F. Del Ponte” Hospital, Varese, Italy. Data were extracted from medical records of two populations of VLBW infants, born in two different timeframes (September 2017 – December 2019 and March 2020 – December 2021). The latter was characterised by both the introduction of a donor human milk bank at the NICU of “F. Del Ponte” Hospital and the beginning of the COVID-19 pandemic. Patients born in 2017 were considered in this analysis only if they were born from September onwards when all NICU cots were converted into single rooms.

Patients weighing < 1500 g were included in the analysis. We excluded those with clinical conditions which might have affected breastfeeding, such as severe congenital malformations, major surgery and severe gastrointestinal disorders, and intraventricular haemorrhage > grade 2. In addition, infants whose mothers could not breastfeed due to other medical conditions or pharmacological treatment not compatible with breastfeeding were also excluded. Donated human milk was given according to the Italian national guidelines [14].

Data regarding type of feeding, the start of minimal enteral feeding, time of FEF achievement, parenteral nutrition duration, growth velocity and length of NICU stay were analysed. Infants were given expressed maternal milk as the first choice, and pasteurised donated human milk if maternal milk was not available.

Achievement of FEF was considered to be at 150 mL / kg / day. Unless otherwise stated, classification of feeding was made according to the predominant type of feeding. We also looked at the 24 hours before and after FEF achievement to check if there were significant discrepancies in the type of feeding, but none were found in our cohort.

For its proven reliability in VLBW infants, we used an exponential regression model to calculate the daily growth rate from the recovery of birth weight until discharge as follows:

$\left[1000\times\;\ln\;\left({\mathrm W}_2\;/\;{\mathrm W}_1\right)\rbrack\;\;/\;\left({\mathrm D}_2\;-\;{\mathrm D}_1\right)\right]$  where W = weight in grams; D = day; 1 = beginning of the time interval and 2 = end of the time interval [15].

Optimal growth was defined as weight gain of more than 17 g / kg / day [16].

As part of the Unit policy, all mothers have been given the opportunity to be supported by our NICU psychologist through individual consultations. The need for support and duration of follow-up were collected.

We evaluated the impact of COVID-19 pandemic related stress considering the need for psychological support requested by mothers and duration of mothers’ follow-up compared to those of babies born before the pandemic period.

Mothers with previously known psychiatric conditions were not considered for this analysis.

## Statistical analysis

All variables have been summarized with absolute and relative frequency, median and interquartile range (IQR). To compare differences between the two groups we applied Chi-square or Fisher exact test for categorical variables and, due to lack of normality distribution, the Mann-Whitney test for continuous ones. Normality was verified using Shapiro-Wilks test and Q-Q plot. Statistical analysis has been done by SAS (version 9.4). Statistical significance was considered when *p* < 0.05.

## Results

A total of 168 infants were analysed: 101 infants were born in 2017 – 2019 and 67 infants were born in 2020 – 2021. Their demographic characteristics are shown in Table 1.

**Table 1:** Demographic data of very low birth weight infants in two time periods

	2017 – 2019 (***n*** = 101)	2020 – 2021 (***n*** = 67)	***P***-value
**Gender –** *n* (%)			
Male	57 (56.4)	33 (49.3)	0.36
Female	44 (43.6)	34 (50.8)	
**Twins** – n (%)	24 (23.8)	24 (35.8)	0.09
**Gestational age (weeks)** – *n* (%)			
Extremely preterm infants (< 28 weeks)	22 (21.8)	15 (22.4)	0.07
Very preterm infants	58 (57.4)	28 (41.8)	
Moderate to late preterm	21 (20.8)	24 (35.8)	
**Birth weight (g)** median (IQR)	1150 (890-1360)	1200 (820-1410)	0.47
IUGR	19 (19.8)	24 (36.4)	0.02*
AGA *n* (%)	64 (63.4)	35 (52.2)	0.34
SGA *n* (%)	23 (22.8)	21 (31.3)	
LGA *n* (%)	14 (13.9)	11 (16.4)	

*AGA* adequate for gestational age, *IUGR* intrauterine growth-restricted, *LGA* large for gestational age, *SGA* small for gestational age

Minimal enteral feeding was commenced on day 1 after birth (median, IQR 1 – 1) in both groups (*p* = 0.11). Median parenteral nutrition duration was 9 days (IQR 7 – 16) in the 2017 – 2019 group, and 9 days (IQR 7 – 16) in the 2020 – 2021 group (*p* = 0.71). The median time to achieve FEF was 12 days (10 – − 17) in the 2017 – 2019 group and 11 days (9 – 16) in the 2020 – 2021 group (*p* = 0.08).

Growth velocity rate was significantly different between the two groups (*p* = 0.01), with a median (IQR) of 16.3 g / kg / day (14.4 – 19.2) in the 2017 – 2019 group and 15.4 g / kg / day (13.3 – 17.2) in 2020 – 2021 group. Accordingly, the proportion of babies experiencing suboptimal growth was significantly higher in the 2020 – 2021 group compared to the 2017 – 2019 group (64 / 67 vs 82 / 101, 95.5% vs 81.2% respectively, *p* = 0.01). No statistical difference was found regarding the median length of stay (*p* = 0,93), which was 47 days (33 – 70) in the 2017 – 2019 group and 47 (33 – 73) in the 2020 – 2021 group.

The type of feeding was also analysed in the two groups at the achievement of FEF. In the 2017 – 2019 group, given the lack of donor human milk bank milk, data were collected considering the prevalent use (> 50%) of mother’s expressed milk vs infant formula. On the other hand, in the 2020 – 2021 group prevalent maternal expressed own milk use was compared to the use of infant formula and / or donated human milk. Interestingly, the use of infant formula was almost the same between the groups (14 / 101 in the 2017 – 2019 group and 10 / 67 in the 2020 – 2021 group, 13.9 and 14.9% respectively).

**Fig. 1:**
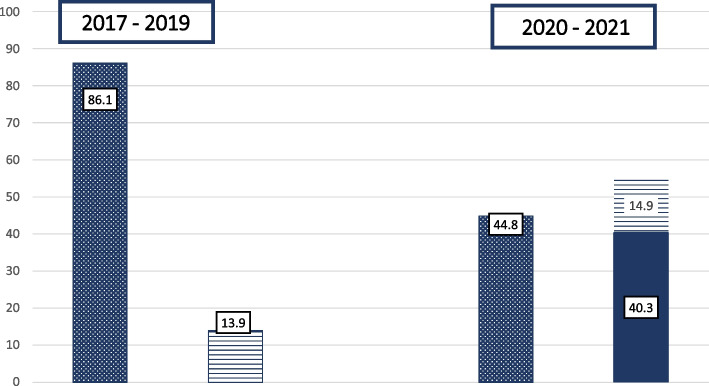
Type of feeding at full enteral feeding achievement in the two groups. Data are expressed as %. Dotted columns indicated infants fed with prevalent maternal expressed milk and lined columns indicated those fed with infant formula. Full coloured column in the 2020-2021 group indicates infants fed with donated human milk

In fact, among the infants who did not receive their own mothers’ expressed milk in the pandemic group (37 / 67, 55.2%), 27 / 67 infants (40.3%) received donor human milk bank milk. Results are outlined in Fig. 1, showing a significant difference between the groups (*p* < 0.001) in the availability of expressed maternal milk.

**Fig. 2:**
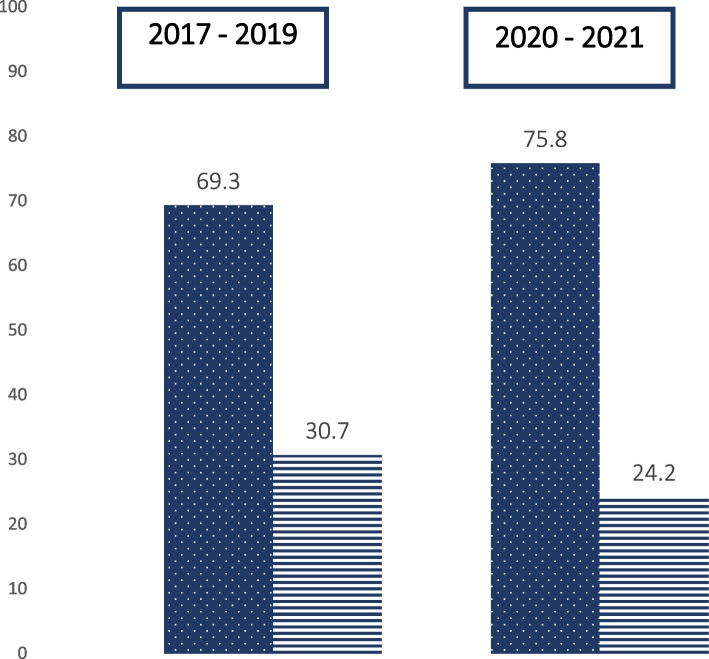
Type of feeding at discharge in the two groups. Data are expressed as %. Lined columns indicate exclusive breastfeeding and the dotted columns indicate all other types of feeding

The same analysis was performed at discharge, where the difference was not statistically significant anymore between the two groups. Maternal milk, exclusive or in combination with other types of milk, was the predominant feeding in almost the same proportion of babies (74 / 101 in the 2017 – 2019 group vs 48 / 67 in the 2020 – 2021 group, 73.3% vs 72.2% respectively, *p* = 0.94). The exclusive breastfeeding rate was not statistically different between the two groups either (31 / 101 in the 2017 – 2019 group vs 16 / 67 in the 2020 – 2021 group, 30.7% vs 24.2%, respectively, *p* = 0.36). Results are outlined in Fig. 2.

No statistical differences were reported in the main clinical outcomes (Table 2). Psychological support was offered to all mothers during the NICU stay.

**Table 2:** Main complications in very low birth weight infants in the two time periods

	2017 – 2019 (***n*** = 101)	2020 – 2021 (***n*** = 67)	***P***-value
**PDA** – *n* (%)	23 (22.8)	22 (32.8)	0.15
**BPD** – *n* (%)	24 (23.8)	15 (22.4)	0.84
**Sepsis** – *n* (%)	6 (5.9)	4 (6.0)	0.99
**Cholestasis** – *n* (%)	6 (5.9)	2 (3.0)	0.48
**ROP grade > grade 2** – *n (%)*	15 (14.9)	7 (10.5)	0.41
**IVH grade 1-2, cerebellar haemorrhage** – *n(%)*	13 (12.9)	13 (19.4)	0.25

*BPD* bronchopulmonary dysplasia, *IVH* intraventricular haemorrhage, *PDA*patent ductus arteriosus, *ROP* retinopathy of prematurity

Thirty-three mothers (33%) in the 2017 – 2019 group and 43 mothers (64%) in the 2020 – 2021 group asked for psychological support (*p* < 0.001). Regarding duration, one mother was followed up by the NICU psychologist for more than six months (1%) in the 2017 – 2019 group, compared to 10 mothers (15%) in the 2020 – 2021 group (*p* < 0.001).

## Discussion

To our knowledge, this is the first study on the impact of the COVID-19 pandemic on the availability of expressed maternal milk in NICU with a further analysis on the role of a donor human milk bank.

One of the most relevant findings of our study is the marked decrease of available expressed maternal milk in our NICU during the pandemic. A recent study by Gunes and co-workers showed that the parent’s breast milk delivery to NICU decreased during the pandemic, even if the exclusive breastfeeding rate at discharge remained unchanged [17]. These findings are consistent with ours. In addition, our results valued the presence of donor human milk bank milk use in the NICU. In our 2020 – 2021 group, in fact, the availability of donated human milk seemed to have highly mitigated the use of infant formula. Donated human milk, accounted for 40.3% of feeds, which is basically the difference between the availability of expressed maternal milk during the pre-pandemic and pandemic period. Although there is evidence that the presence of a donor human milk bank favourably correlates with the breastfeeding rate [18] in NICU, the pandemic period during which it was opened, is likely to have markedly reduced its potential.

An explanation for the lack of significant differences in terms of main clinical outcomes might partly be found in such use of donated human milk. First, no significant differences were found in the time to achieve FEF and in the parenteral nutrition duration, similar to findings of other authors [19]. Then, it is widely known that human milk contains bioactive components and microorganisms that shape the development of the intestinal microbiota contributing to improved clinical outcomes [1, 20]. The population we examined was particularly at risk for complications related to prematurity, which did not increase during the pandemic, despite the reduced use of expressed maternal milk. Given the same level of care provided in our NICU and the same single room NICU setting in the two timeframes analysed, we may speculate that donated human milk contributed to maintaining the clinical outcomes within our previous standards.

Moreover, we showed that the rate of exclusive breastfeeding as well as the rate of formula-fed babies were not statistically different between the two groups at discharge. This might be due to several factors. First, our medical and nursing staff is particularly dedicated to promoting breastfeeding, and this is shown by the high rate of breastfeeding in the 2017 – 2019 group. Moreover, the presence of a donor human milk bank, reducing the initial frustration and stress due to preterm birth and related difficulties, might have promoted the subsequent establishment of breastfeeding [21, 18]. Then, the increasing evidence supporting the practice of breastfeeding during the pandemic has contributed to reassuring mothers regarding the safety and value of their milk. The initial conflicting evidence on the feasibility of breastfeeding at the time of the COVID-19 pandemic, together with uncertainty regarding the viral transmission through maternal milk, has certainly discouraged mothers in the early stage of this emergency [13]. To date, there is indeed convincing evidence from the leading authorities that the use of expressed milk is safe and highly recommended [22].

The significantly lower growth rate between the two groups might be explained by a significantly higher number of intrauterine restricted infants in the 2020 – 2021 group. Also, our findings confirmed what is already known about the different compositions of donated milk and maternal milk and its impact on preterm infants’ growth rates [23, 4].

The daily availability of a dedicated psychologist in NICU allowed us to both provide support to mothers and better interpret our findings. Although the effects of the pandemic on maternal stress are now well known [10], there is little evidence so far that it has impacted the rate of expressed milk in the NICU.

The parental stress related to the pandemic is well documented [12, 10, 11] so far, as well as the stress related to recovery in NICU and its influence on breastfeeding [7–10]. Our data confirmed that the stress level in mothers was higher than before the pandemic, with a significantly higher need for psychological support and more prolonged follow-up. During the pandemic, restrictive measures have been taken in our hospital regarding access to the NICU. At the onset of the pandemic, only one visitor at a time was admitted, so alternating visits by mothers and fathers were allowed. During the subsequent pandemic waves, further restrictions were introduced and only mothers were admitted to the NICU, with fathers being excluded from visiting their babies. The inability to share the emotional difficulties related to the NICU recovery with their partners, in addition to the fear related to the pandemic period, have reasonably been supposed to have increased the stress of mothers significantly. Consequently, due to the strict interplay between stress and effective breastfeeding [24, 25], maternal expressed milk availability dramatically decreased. Moreover, since the role of fathers in NICU in promoting breastfeeding has been well described [26–28], it is reasonable that their absence might have played an additional significant role in reducing maternal milk expression.

The role of counselling and support during breastfeeding in NICU has been widely demonstrated, and this might further explain why the breastfeeding rate at discharge rose again, reaching the previous level [29–31].

Our study has some limitations. First, it is a retrospective study involving a single NICU. Also, at the time of the study, the donor human milk bank had just been opened at our NICU, so it might be possible that this “running-in” stage has minimally influenced our data. Then, given the high rate of breastfeeding in our unit out of the pandemic period, we could not perform any comparison with formula-fed babies, which account for a limited number in our cohort. Last, we were not able to make any consideration regarding the effect of the COVID-19 pandemic on donated human milk availability since the opening of our bank coincided with the pandemic onset.

## Conclusion

Our study showed how the pandemic-induced stress and the related restrictions introduced in a NICU had a significant impact on the availability of expressed maternal milk in that NICU. However, the rate of breastfeeding at discharge was the same as before the pandemic. Therefore, the presence of a donor human milk bank was fundamental to minimise the use of infant formula in very low birth weight infants.

Further research would be desirable to investigate how the use of donated human milk bank milk can be implemented in emergency situations and its impact on clinical outcomes for neonates.

## Data Availability

Data are available upon reasonable request.

## Abbreviations

AGA: *Adequate* for gestational age
BPD: *Bronchopulmonary* dysplasia
FEF: *Full* enteral feeding
IUGR: *Intrauterine* growth-restricted
IVH: *Intraventricular* haemorrhage
LGA: *Large* for gestational age
NICU: *Neonatal* intensive care unit
PDA: *Patent* ductus arteriosus
ROP: *Retinopathy* of prematurity
SGA: *Small* for gestational age
VLBW: *Very* low birth weight
